# Implementing advance care planning with people from ethnic minority backgrounds with cancer: a qualitative study of factors affecting practice in Australia

**DOI:** 10.1007/s00520-025-09707-z

**Published:** 2025-07-01

**Authors:** Ashfaq Chauhan, Ursula M. Sansom-Daly, Elizabeth Manias, Margo Van Poucke, Mashreka Sarwar, Nyan Thit Tieu, Nadine El-Kabbout, Ramya Walsan, Upma Chitkara, Misbah Faiz, Vitor Moraes Rocha, Reema Harrison

**Affiliations:** 1 Centre for Health Systems and Safety Research, Australian Institute of Health Innovation, Macquarie University, North Ryde, NSW Australia; 2 School of Clinical Medicine, Discipline of Paediatrics & Child Health, Behavioural Sciences Unit, UNSW Medicine and Health, Randwick Clinical Campus, UNSW Sydney, Randwick, NSW Australia; 3 Kids Cancer Centre, Sydney Children’s Hospital, Randwick, NSW Australia; 4 Sydney Youth Cancer Service, Nelune Comprehensive Cancer Centre, Prince of Wales Hospital, Randwick, NSW Australia; 5 School of Nursing and Midwifery, Monash University, Melbourne, VIC Australia; 6 School of Education, Macquarie University, North Ryde, NSW Australia; 7 Sisters’ Cancer Support Group Inc, Mount Pleasant, NSW Australia; 8 Nafs Counselling, Sydney, NSW Australia; 9 Health Professional Council Authority, Sydney, NSW Australia; 10 Murray Primary Health Network, Bendigo, VIC Australia

**Keywords:** Advance care planning (ACP), Ethnic minority, Cancer, Equity, Person-centred

## Abstract

**Purpose:**

Despite the availability of resources to support advance care planning (ACP) among people from ethnic minority backgrounds, its uptake remains low among these communities presenting an ACP implementation gap. This study was conducted to understand barriers and facilitators in service delivery experienced by healthcare staff and interpreters involved in implementing ACP with people from ethnic minority backgrounds with cancer.

**Methods:**

A qualitative study using focus groups and semi-structured interviews was conducted with eligible healthcare staff and interpreters. Data was analysed employing the Framework Method using the Theoretical Domains Framework to understand ACP implementation factors.

**Results:**

Eight focus groups comprising 28 participants and three individual semi-structured interviews were conducted. Four themes were developed, along with an underpinning theme of interprofessional collaboration between clinicians and interpreters in ACP. The four themes were as follows: (1) skills of the healthcare clinicians and interpreters; (2) knowledge of cultural factors that impact ACP; (3) the care setting and the physical environment for ACP; and (4) availability of resources to support ACP. Opportunities to foster interprofessional collaboration between clinicians and interpreters, such as training in working with each other and briefing and debriefing, were not available.

**Conclusions:**

Shared understanding between interpreters and cancer clinicians about their communication approach and terminologies to use when no direct translation is available may contribute towards increased uptake of ACP. Identification and necessary adaptations of mechanisms that foster interprofessional collaboration in cancer care between clinicians and interpreters in ACP communication with ethnic minority communities will enhance ACP uptake and person-centred care.

**Supplementary Information:**

The online version contains supplementary material available at 10.1007/s00520-025-09707-z.

## Introduction

Advance care planning (ACP) is a process in which a person’s future health care needs, wishes and preferences are communicated so that these could be acted upon at a time when they are unable to make the relevant decisions by themselves [1, 2]. ACP communication may involve, but is not limited to, discussions around a person’s preferred health or medical care at the end-of-life, place for end-of-life care, or completing an advance care directive or appointing a substitute decision-maker [2, 3]. The presence of ACP is associated with care that aligns with patient preferences, contributing to enhanced health and psychological outcomes for patients, their family and healthcare staff [3]. In Australian context, people from ethnic minority backgrounds include those being born overseas, who have one or more parents born overseas or who speak a language other than English at home [4]. People from ethnic minority backgrounds experience inequity in end-of-life related health outcomes. For example, they experience poor pain assessment and management, and are more likely to receive intensive care at the end-of-life [5, 6].

Cancer can be a life-limiting illness; ACP is important in cancer due to many reasons including complex treatment regimen, rapid disease progression and frequent change in treatment planning [7]. ACP is particularly important for people from ethnic minority backgrounds with cancer due to potential for differences in perceptions of cancer and expectations of treatment. Some ethnic minority communities believe cancer to be a shameful disease or have fatalistic views about its diagnosis [8], while some wish to not talk death and dying [9, 10], therefore preventing these communities to engage meaningfully in ACP. ACP communication with people from ethnic minority backgrounds is also impacted by differences in principles of individual autonomy and in collectivist cultures with family-oriented decision-making [10]. Families’ desire to not disclose diagnosis of cancer or prognosis as it may be emotionally harmful to the patient may impact meaningful involvement of ethnic minority communities in ACP [9, 11–13]. Some ethnic minority communities may be misled by medical terms used in oncology and palliative care even when interpreters were used [14], potentially constraining ACP communication.

Many resources in form of printed documents and videos have been produced internationally by health systems and services to support uptake of ACP among ethnic minority communities [2, 15, 16]. These resources aim to raise awareness and knowledge of ACP among people from ethnic minority backgrounds and their clinicians, and provide structured guidance on how to think about, initiate or conduct ACP [16, 17]. Despite the availability of resources, ACP uptake among people from ethnic minority backgrounds remains low [18], presenting an ACP implementation gap.

Reasons for the low ACP uptake among ethnic minority communities appear to relate to low confidence among clinicians about initiating ACP communications with these communities [18]. Interpreters are also required to conduct ACP with people from ethnic minority backgrounds who need language assistance. Data suggest a lack of support for interpreters involved in ACP discussions in cancer care, and limited guidance about how interpreters can be involved in ACP to enable these conversations [16, 19]. There has been limited exploration of experiences of healthcare staff and interpreters of factors that contribute to ACP implementation with people from ethnic minority backgrounds with cancer in Australia [14, 20]. This study aimed to identify barriers and facilitators in service delivery experienced by healthcare staff and interpreters involved in implementing ACP with people from ethnic minority backgrounds with cancer using the Theoretical Domains Framework (TDF) [21–23].

## Method

### Ethics

Ethical approval was granted by the Macquarie University Human Research Ethics Committee (Ref: 520,231,235,452,528). The study was conducted in accordance with the National Statement on Ethical Conduct in Human Research [24].

### Design

A cross-sectional, qualitative, exploratory study using focus groups was conducted. Semi-structured interviews were conducted with participants who were unable to attend a focus group discussion. Consolidated Criteria for Reporting Qualitative Studies guidelines were used to report this study (Supplementary File 1) [25].

### Setting

Delivery of healthcare is a shared responsibility between the federal and states or territories health departments in Australia with ACP policies, practices and responsibilities intersecting between the two. Considering this, nationwide focus groups or semi-structured interviews were conducted with eligible participants based on their availability.

### Recruitment

The recruitment was purposive, targeting participants from different professions (doctors, nursing, allied health), settings (inpatient, community, outpatient) and state/territories. Concept of information power was used to determine the sample size by employing an iterative process to recruit participants across state/territories from diverse professional expertise, roles and language expertise [26]. Participants aged 18 years or older, working in cancer or palliative care services for a minimum of 6 months, in clinical (e.g. medical doctors, allied health and nursing) or non-clinical (e.g. managers and administrative staff) roles as well as interpreters were eligible. To recruit healthcare staff, study advertisement was distributed through cancer and palliative care member organisations, care networks, communities of practice, members of the project steering group and through social media posts. To recruit professional interpreters, study advertisement was distributed via a member of the project steering group, a national interpreter agency and a national professional interpreter association. The study advertisement included the contact details of two researchers (AC, RH). Interested participants contacted the researchers and were provided with participant information sheet and consent form. Written consent was obtained from each participant via email prior to the data collection.

### Study material and data collection

A topic guide was developed by two researchers (AC, RH) based on the TDF to support focus groups and semi-structured interviews. Feedback on the topic guide was received from 10 members of the Project Steering Group, including three consumers from ethnic minority backgrounds with lived experience of cancer. Data were collected by a clinical physiotherapist and health services researcher (AC) using online videoconferencing between March 2023 and May 2024. Data were recorded, transcribed verbatim and anonymised.

### Data analysis

Data were inductively and deductively analysed using the Framework Method of analysis grounded in the TDF (Supplementary File 2) [27]. Data were first inductively coded into barriers and facilitators for ACP implementation among ethnic minority consumers with cancer. These barriers and facilitators were grouped into categories, which were then deductively mapped against the TDF domains and resulting themes were formed. Supplementary file 2 describes the process of development of themes.

After transcription, two researchers, one physiotherapist (AC) and one linguist (MVP), familiarised themselves with the data by re-reading the transcripts several times. Following this, three transcripts were independently coded by the two same researchers. The two researchers met to compare their findings and develop a working analytic framework. These two researchers then independently completed the coding of the remaining transcripts. During this process, they met every fortnightly to discuss their findings and update the working analytic framework. The findings were also discussed with project lead (RH) in separate fortnightly meetings and the framework was refined. Following this, a Framework Matrix charting and final codes were developed (Supplementary File 2) [27]. A subset of transcripts were also reviewed by other team members (MS, EM, UMS-D, NTT) and findings discussed. Preliminary themes were then identified which were further refined through discussion between research team members. This process contributed to study rigour.

## Findings/results

### Participant characteristics

Eight focus groups (28 participants) and three individual semi-structured interviews were conducted, totalling 31 participants. Table 1 describes the participant characteristics against the participant codes. In reporting the results, the term staff is used to describe both clinicians in patient-facing roles (nurses, medical doctors, and allied health) and healthcare managers. Main quotes supporting the themes are provided in tables under respective theme.

**Table 1:** Participants code, professional background and location across Australia

Participant Code	Profession	Role	State
P1_FG1	Nurse	Palliative care nurse practitioner	Western Australia
P2_FG1	Doctor	Palliative care registrar	New South Wales
P3_FG1	Nurse	Palliative care nurse consultant with background in cancer care	Victoria
P4_FG1	Allied health	Social worker	New South Wales
P5_FG1	Healthcare manager	Healthcare manager	Victoria
P1_FG2	Allied health	Social worker	Victoria
P2_FG2	Allied health	Pastoral care	New South Wales
P3_FG2	Nurse	Cancer care registered nurse	New South Wales
P4_FG2	Nurse	Palliative care nurse consultant with background in cancer care	New South Wales
P1_FG3	Nurse	Palliative care registered nurse	New South Wales
P2_FG3	Allied health	Social worker	Western Australia
P4_FG3	Healthcare manager	Healthcare manager	Western Australia
P1_FG4	Nurse	Cancer care registered nurse	South Australia
P2_FG4	Allied health	Social worker	Queensland
P3_FG4	Nurse	Palliative care nurse practitioner	Queensland
P1_FG5	Nurse	Palliative care nurse manager	South Australia
P2_FG5	Allied health	Social worker	South Australia
P3_FG5	Allied health	Social worker	South Australia
P4_FG5	Nurse	Palliative care nurse practitioner	New South Wales
P1_FG6	Doctor	Medical oncologist	New South Wales
P2_FG6	Doctor	Palliative care consultant	New South Wales
P1_FG7	Doctor	Medical oncologist	South Australia
P2_FG7	Doctor	Palliative care specialist	New South Wales
P3_FG7	Healthcare manager	Healthcare manager	Victoria
P4_FG7	Doctor	Medical oncologist	New South Wales
P1_FG8	Interpreter	Polish Interpreter	Victoria
P2_FG8	Interpreter	Greek Interpreter	South Australia
P3_FG8	Interpreter	Filipino Interpreter	Victoria
P1_SI	Interpreter	Japanese Interpreter	Queensland
P1_SI	Interpreter	Hazara/Pashto Interpreter	Victoria
P1_SI	Interpreter	Mandarin interpreter	Victoria

*P*: Participant, *FG*: Focus group, *SI*: Semi-structured interview

### Key themes

**Fig. 1:**
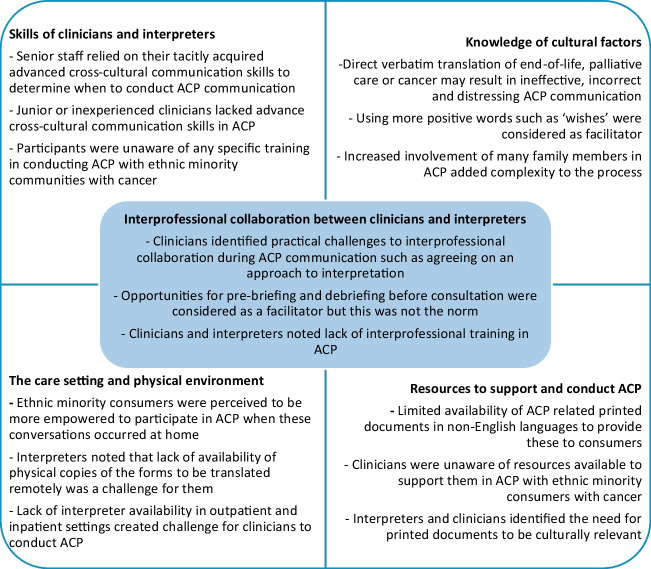
Factors impacting implementation of advance care planning with people from ethnic minority backgrounds with cancer

Participants characterised high-quality ACP as a shared decision-making process that occurs throughout a person’s care and provides them with options, underpinned by the principles of trust, honesty, truthfulness and openness. Participants said these underlying principles of ACP were similar for both people from ethnic minority and non-ethnic minority backgrounds with cancer, but remarked that their implementation varied between the two groups. Participants acknowledged that ACP implementation with people from ethnic minority backgrounds with cancer was a complex process impacted by a range of factors. Using the TDF as a guide to understand the barriers and facilitators for implementation of ACP, four interrelated themes were developed with an underlying theme of interprofessional collaboration between clinicians and interpreters (Fig. 1). These four themes were as follows: (1) skills of clinicians and interpreters, (2) knowledge of cultural factors that impact ACP, (3) the care setting and the physical environment for ACP, and (4) availability of resources to support and conduct ACP.

### Skills of clinicians and interpreters

#### Dynamic ACP communication requires advanced cross-cultural communication skills

ACP was considered to be a dynamic process where these conversations could occur at any time. Noting this, clinicians described that along with general cross-cultural communication skills (such as talking at an 8-year-old level or using an interpreter), relying on both verbal and non-verbal cues from people from ethnic minority backgrounds with cancer to determine when to start or stop ACP conversations was important for conducting these conversations (Table 2, Nurse_P3_FG2).

**Table 2:** Main quotes for theme 1 “skills of clinicians and interpreters”

Participant	Quote
Nurse_P3_FG2	“you get verbal and non-verbal cues about, okay, it looks like this is enough now. We'll stop. Or you change track. I think that comes with experience.”
Allied_health_P4 _ FG2	“if it was a JMO (junior medical officer), I would tend to lead that conversation….and just allow them to watch and listen, about how we actually phrased things.”
Nurse_P3_FG2	“So many people didn’t have a great understanding [of advance care planning], because interpreters were not used.”
Doctor_P2_FG1	“I guess the need for training [in ACP] goes for both parties [clinicians and interpreters].”
Nurse_P1_FG5	“I had a case where mum and daughter disagreed. So, then we had an interpreter who was kind of picking sides almost.”
Interpreter_P3_FG8	“we [interpreters] get into the room not knowing, you know what the situation is and then you have all those emotions happening around.”
Nurse_P4_FG2	“I will often tell the interpreter that [type of consultation] before we get into it, so that they're aware that they're going to be managing this type of conversation.”
Doctor_P4_FG7	“it would be helpful if interpreters were debriefed, …. because in terms of formal training for oncology or palliative care type discussions., there's not very much. So, it can be quite confronting.”

Clinicians mentioned that they often acquired these cross-cultural communication skills through tacit learning over time. As a result, these skills were perceived to be lacking among more junior or new clinicians. This was identified as a challenge for junior or new clinicians to adapt to dynamic ACP communication processes, with senior clinicians often required to lead these conversations (Table 2, Allied_health_P4 _ FG2).

#### Interprofessional collaboration between clinicians and interpreters is essential

Interpreters described themselves as passive conduits and as being outside of the care team. Clinicians also thought that the task of repeating the information when interpreters were used may make the ACP communication less therapeutic and disrupt flow of conversation due to repetition. However, both clinicians and interpreters identified that partnership between them was necessary for more effective ACP (Table 2, Nurse_P3_FG2).

Participants highlighted that they were not aware of any training in ACP communication with people from CALD backgrounds available to them. Clinicians particularly identified the need for two-way training in how to conduct ACP conversations when interpreters were used (Table 2, Doctor_P2_FG1).

Clinicians identified practical challenges to interprofessional collaboration during ACP communication such as agreeing on the general approach together, as well as more specifically the approach to managing family expectations when interpreters were used during ACP communication. A clinician also provided example where an interpreter was visibly distressed during ACP communication, where distress was attributed to the lack of knowledge about nature of the consultation. Interpreters also felt that they were unsupported and unprepared for ACP communication due to lack of knowledge beforehand about the nature and purpose of the consultation (Table 2, Nurse_P1_FG5; Interpreter_P3_FG8).

Noting the sensitive nature of the communication, clinicians and interpreters verbalised the importance of briefing (a short meeting between interpreter and clinician immediately before the consultation) and debriefing (a short meeting between interpreter and clinician immediately after the consultation). They said that briefing and debriefing helped to clarify the nature and the expectations of consultation, and offer support to interpreters after the consultation if needed. However, both briefing and debriefing were described as not being the norm and were seen to be dependent on an individual clinician’s or interpreter’s unique ways of working or practising (Table 2, Nurse_P4_FG2; Doctor_P4_FG7).

### Knowledge of cultural factors that impact ACP

#### Verbatim interpretation inhibiting ACP

Clinicians and interpreters mentioned that direct verbatim translation of terms such as end-of-life care, cancer and palliative care was not always possible or desirable. Verbatim translations of these concepts may result in incorrect, ineffective and distressing ACP communication for people from ethnic minority backgrounds with cancer (Table 3, Nurse_P4_FG5).

**Table 3:** Main quotes for theme 2 “knowledge of the cultural factors that impact ACP”

Participant	Quote
Nurse_P4_FG5	“Certainly, the terms for Chinese for palliative care is incorrect. and then there is what the interpreters use as well because they are bound by the health to use those terms.”
Interpreter_P2_FG8	“there are those isolated people who say just interpret verbatim.”
Nurse_P4_FG2	“so, I'll often ask [to patient], what's culturally acceptable for you and your family, in terms of what words we choose to use?”
Doctor_P2_FG1	“I like to start by getting everyone in one room in an open discussion with the patient, and their loved ones…. ask them to nominate one person.”
Doctor_P4_FG7	“They [interpreters] can provide their own cultural insights if they happen to be of a similar cultural background.”
Doctor_P1_FG6	“if your family member is saying a certain thing, are you happy for them to be speaking for you on those matters… Just so that it's quite clear who can and can't be saying certain things on their behalf.”
Interpreter_P3_FG8	“You know you have many hats [also a community leader], in that situation [so] you're conscious of the limitations of your role.”
Interpreter_P1_FG8	“I'm here to do interpreting in your language that everything's confidential.”

A Mandarin-speaking nurse noted that the verbatim translation of palliative care was “giving up.” Another nurse gave the example of cancer being translated as “God forbid” in Arabic. In the Greek language, an interpreter noted that palliative care was purposefully translated to “respite care.” Participants, especially interpreters, mentioned that such verbatim translation did not reflect the true meaning of ACP concepts. Thus, request for and expectations of verbatim translations from clinicians only acted as a barrier for meaningful ACP communication (Table 3, Interpreter_P2_FG8).

#### Adapting ACP communication to meet cultural needs

Considering the importance for maintaining hope during ACP, participants noted importance of using alternate words that were considered more welcoming and positive. One palliative care nurse said that they use words “wishes” and “preferences” instead of “end-of-life planning” during ACP with people from ethnic minority backgrounds with cancer. Clinicians also asked people from ethnic minority backgrounds with cancer before the appointment to determine and share what terms were both acceptable in their culture, and to them, personally. Clinicians also verbalised that interpreters could provide cultural insight to them before ACP communication; however, this was not commonly practised (Table 3, Nurse_P4_FG2; Doctor_P4_FG7).

#### Complexity of family-based decision-making

Increased family involvement in ACP for people from ethnic minority backgrounds with cancer added complexity to ACP communication, articulated mainly due to family-oriented decision-making approaches adopted in some cultures. In instances in which a family requested not to disclose the diagnosis of cancer or prognosis to the patient or when multiple family members were involved in ACP, clinicians were tasked with navigating differing wishes and unable to determine what the patient wanted. To manage these barriers, clinicians used approaches such as providing opportunities to the patient to ask questions during the consultation, agreeing on one decision-maker, and meeting with patients and interpreters to confirm that patients agreed to allow an appointed decision-maker to make decisions on their behalf (Table 3, Doctor_P2_FG1; Doctor_P1_FG6).

#### Interpreters’ role in the community and ACP communication

Interpreters, who were also often community leaders, expressed limitations of their role in ACP whilst also carrying responsibilities as a community leader as some consumers from ethnic minority backgrounds with cancer sought advice for ACP or asked direct questions to them. To manage this advice giving, interpreters used non-verbal cues and hand gestures during ACP conversations to direct the patient or their family to ask questions directly to clinicians (Table 3, Interpreter_P3_FG8).

Clinicians and interpreters noted that some consumers from smaller ethnic minority communities may not wish to involve interpreters as they may not want to reveal their diagnosis of cancer or prognosis to someone they know. In such circumstances, interpreters highlighted the importance of reinforcing the confidential nature of the appointment (Table 3, Interpreter_P1_FG8).

### The care setting and the physical environment for ACP

#### Consultations at home encouraged open communication in ACP

Clinicians said that people from ethnic minority backgrounds with cancer appeared more empowered to participate in ACP when these conversations were conducted in their home as compared to in a hospital setting. They attributed this to the notion that the clinician is a guest in their home environment, reducing the power differential. This was deemed particularly useful when commencing ACP communication (Table 4, Nurse_P3_FG1).

**Table 4:** Main quotes for theme 3 “the care setting and the physical environment for ACP”

Participant	Quote
Nurse_P3_FG1	“[At home] he could express himself [in ACP], and I was the guest…I think people, once they [patient] get to hospital, feel that loss of power.”
Allied_health_P2_FG5	“[I] have time to establish [the/a good] relationship most of the time, so I can always do some background [work to] gain the trust and then go back again.”
Doctor_P1_FG6	“There's often more family around so more space, more time, because communication often takes longer. You have to just go over things several times to check. check back in all the time that they're understanding what you're saying.”
(Healthcare_manager_P5_FG1)	“we don't have level 3 [certified] interpreters in all languages, and there's a fluctuation in interpreting and interpreters in every language.”
Doctor_P2_FG6	“[It can be really hard if] the phone line's really crackly or if a family meeting goes for more than an hour, which they can do, [and then] the phone [automatically] cuts off at 60 min.”
Interpreter_P1_FG8	“These [resuscitation] forms are not translated, and we're not given those forms ahead of time.”

Clinicians also noted that conducting ACP communication over multiple visits at the place of residence of people from ethnic minority backgrounds with cancer allowed them to build trust and relationship with them, facilitating ACP communication (Table 4, Allied_health_P2_FG5).

When ACP occurred in hospital settings, the setup of the room and time allocated for appointment were identified as factors contributing to ACP implementation. Conducting ACP in a shared hospital room with loud noises was perceived as a barrier due to private and sensitive nature of the communication. Recognising the involvement of multiple family members in ACP for people from ethnic minority backgrounds with cancer, it was considered important to have a spacious room where everyone could sit comfortably. However, a common barrier experienced by clinicians while conducting ACP at home or in hospital was the consistent lack of qualified interpreters in some languages (Table 4, Doctor_P1_FG6).

#### Suboptimal interprofessional collaboration during remote ACP

Clinicians and interpreters noted the difficulty of conducting ACP via remote consultation (when interpreters attend consultation over the phone). In addition to lack of non-verbal cues to rely on, participants noted various technical and logistical issues that impacted ACP with people from ethnic minority backgrounds with cancer (Table 4, Doctor_P2_FG6).

Interpreters particularly stated difficulty in translating forms (such as Resuscitation Forms) over the phone, verbally and in “real time,” as they did not have a physical copy of the form with them. They voiced the importance of receiving such forms ahead of the remote consultation as imprecise translation may lead to wrong decisions being made (Table 4, Interpreter_P1_FG8).

### Availability of resources to support and conduct ACP

#### Lack of ACP resources in non-English languages

Resources for ACP, such as Advance Care Directives and ACP booklets, were variously made available to consumers from ethnic minority backgrounds with cancer. Availability of these resources across services varied as some clinicians mentioned that they were available but not in all non-English languages while other mentioned that they did not have access to the physical copies of these resources in their cancer service (Table 5, Doctor_P2_FG7).

**Table 5:** Main quotes for theme 4 “availability of resources to support and conduct ACP”

Participant	Quote
Doctor_P2_FG7	“I don't think we have any physical copies within our cancer centre.”
Doctor_ P1_FG6	“If you don't have an interpreter handy, and you can't explain the document to them or go through it with them which is challenging.”
Interpreter_P1_FG8	“They are continuing translating palliative care as respite care (in printed resources) … because if you think about it, say, a palliative care nurse turns up to your home and you think it's a respite.”

Clinicians also added that providing instructions for how to use the ACP documents were important, but this was contingent on availability of interpreters. This was a challenge as trained interpreters were not available in all the languages or dialects consistently (Table 5, Doctor_ P1_FG6).

Interpreters also noted that at times, the verbatim translations or words such as “palliative care” in the ACP and other palliative care documents for consumers from ethnic minority backgrounds was incorrect. Interpreters raised concerns as the use of incorrect terms may create confusion between the consumers from ethnic minority backgrounds and clinicians on the purpose of ACP (Table 5, Interpreter_P1_FG8).

## Discussion

Applying the TDF, the study identified a range of barriers and facilitators that influenced ACP implementation for people from ethnic minority backgrounds with cancer. These barriers and facilitators spanned initiation to communication throughout ACP. Key barriers were related to unequal power dynamics between clinicians and consumers from ethnic minority backgrounds with cancer in certain settings, limited skills in addressing ACP among clinicians and lack of opportunities for interprofessional collaboration between clinicians and interpreters.

Recent systematic review has highlighted lack of awareness of ACP among ethnic minority communities as a barrier to uptake of ACP among these communities [18]. Educational interventions targeting these communities have documented improved knowledge and awareness of ACP among them, but have not translated into greater ACP uptake [28]. Our findings that people from ethnic minority backgrounds with cancer may feel less empowered to conduct these conversations in some settings (such as hospitals) may indicate that along with knowledge and awareness of ACP, equal power dynamics are needed for meaningful engagement of these communities in ACP [29, 30].

Lack of cross-cultural communication skills among junior or new clinicians for when to initiate and communicate about ACP was identified as a barrier for implementing ACP with people from ethnic minority backgrounds with cancer. International evidence highlights clinicians poor understanding of cultural norms around ACP and lack of skills in communication about ACP with ethnic minority communities as a barrier for ACP implementation among these communities [18, 31]. Experiential learning with senior colleagues in ACP communication may support junior or new staff to build their skills in initiating and communicating about ACP. However, reliance solely on experiential learning may mean that optimal ACP communication may not occur until necessary skills are gained. This may perpetuate further inequity. Experiential learning also requires enablers at service level such as sufficient staffing numbers and ensuring that junior/new clinicians are paired with senior staff. Learning from senior clinicians also warrants further examination to determine which senior clinicians are well equipped to train new clinicians in ACP and if any formal recognition is required.

Lack of interprofessional collaboration between interpreters and clinicians for the purpose of ACP was a critical barrier. Poor interprofessional collaboration between clinicians and interpreters had been discussed as a barrier to uptake of ACP among ethnic minority communities in international studies, conducted with participants from range of settings [19, 32, 33]. A qualitative study of 24 clinicians from the United Kingdom, that included two cancer clinicians, identified that clinicians had no training in working with interpreters for conducting resuscitation discussions with people from ethnic minority backgrounds [32]. Another qualitative study conducted with 20 professional healthcare interpreters assisting in consultations with patients from ethnic minority backgrounds during their transition to palliative care from cancer care identified that interpreters did not feel empowered to collaborate with clinicians [19]. Insufficient collaboration between interpreters and clinicians was described as contributing to miscommunication between clinicians and consumers from ethnic minority backgrounds, along with lack of cultural sensitivity in ACP communications and exposure to emotional trauma among interpreters due to the unexpected and challenging nature of the communication [34].

One of the fundamental challenges for ACP among diverse cultural populations is that ACP has predominantly been developed in a western cultural context [35], which may not be well-aligned with the diverse cultural groups that are served within the Australian health system [3, 9, 36]. However, as a central goal of ACP is to understand and respond to individuals and their wishes, there may be mechanisms for this approach to be applied beyond the cultural contexts in which it has been developed [3]. Recognising ACP as an ongoing communication process, there is a value in applying the principles of patient centred medicine (exploring health, understanding the whole person, finding common ground, and enhancing the patient-clinician relationship) in clinical practice to support ACP communication with people from diverse cultural backgrounds [37].

### Implications

Interprofessional collaboration plays an important role in collaborative practice by supporting three-way communication that foster equal interactional power and shared understanding between clinicians, interpreters and consumers from ethnic minority backgrounds [38–40]. Interprofessional collaboration between interpreters and clinicians may contribute to agreement on approaches to use for communicating ACP information, and terminologies to use when verbatim translation is not possible. One mechanism through which interprofessional collaboration can be enhanced is interprofessional education [34, 38, 41]. To promote adult learning making interprofessional education interactive (such as using problem based learning or role play) is also recommended [42]. An interactive interprofessional education program delivered to interpreters and palliative care clinicians at one hospital in the US demonstrated increased confidence among interpreters for ACP communications [34]. Adaptation to the content and the program process to meet the needs of the local cancer service may be a useful approach.

International practice guidelines, policies and procedures have recommended briefing and debriefing during ACP discussions when interpreters are involved [43–45]. One hospital network in Australia had successfully implemented briefing in their palliative care service for in-person interpreter appointments [46]. Special considerations will be required for implementing briefing/debriefing when interpreters are joining the consultation remotely, as this appears to be more common in the post-COVID environment [47] and when agency interpreters are involved. The study also highlighted the need for providing remote interpreters with necessary ACP documents prior to the consultation for them to be able to convey the correct ACP related information to the person from ethnic minority background with cancer.

## Limitations

The inclusion of range of healthcare staff and interpreters is a strength of this study; however, the clinician participants largely worked in tertiary or specialist setting. The application of the findings to other settings may be limited. The application of evidence-based TDF allowed in-depth exploration of factors but it is likely that use of this framework may have led to the exclusion of other factors that may not fall within the domains. Other allied health staff, such as physiotherapists, were not recruited and it is likely that they may encounter additional barriers for ACP communication with people from ethnic minority backgrounds with cancer. Consumer views were not included in this research, but this is the focus of a dedicated complementary study being carried out within the wider program.

## Conclusions

The study provides new knowledge of the range of barriers and facilitators that contribute to ACP implementation in people from ethnic minority backgrounds with cancer. Interprofessional collaboration between clinicians and interpreters in communicating about the future health care wishes of people with cancer when language was identified as an important contributor. Having a shared understanding of the communication approach and terminologies to use when no direct translation is available will contribute to increased uptake of ACP among people from ethnic minority backgrounds. Identification and adaptations of mechanisms to foster interprofessional collaboration in cancer care between clinicians and interpreters, such as interprofessional education, may enhance ACP uptake among ethnic minority communities contributing towards person-centred care.

## Supplementary Information

Below is the link to the electronic supplementary material.

ESM1 (PDF 449 KB)

ESM2 (DOCX 16.8 KB)

## Data Availability

Please contact the corresponding author (AC) or the project lead (RH) to enquire regarding access to data.
