# Supplementary material for: Implementing advance care planning with people from ethnic minority backgrounds with cancer: a qualitative study of factors affecting practice in Australia
Source: Support Care Cancer. 2025 Jul 1;33(7):648. doi: 10.1007/s00520-025-09707-z (PMC12214012; doi:10.1007/s00520-025-09707-z)
Supplement: Supplementary file 2 — (DOCX 16.8 KB) [file 520_2025_9707_MOESM2_ESM.docx]

**Supplementary File 2: Framework matrix describing the development of final themes using the Framework Analysis method.**

| **Inductive coding to identify barriers and facilitators** | **Mapping against domains of the TDF** | **Deductive coding of subthemes** | **Final themes** |
| --- | --- | --- | --- |
| Process related factors   - Training of healthcare staff and interpreters in working with each other - Opportunities to discuss the nature of consultation beforehand | Skills  Emotion  Social influences | Dynamic ACP communication requires advanced cross-cultural communication skills  Interprofessional collaboration between clinicians and interpreters is essential | **Skills of clinicians and interpreters** |
| Interpersonal factors   - Skills in cross-cultural communication - Interprofessional skills to work together - Emotional impact of ACP communication on interpreters |  |  |  |
| Cultural factors   - Cultural understanding of cancer and ACP - Family involvement in ACP - Cultural accuracy of ACP related translation | Knowledge  Social/profession role and identity | Verbatim interpretation inhibiting ACP  Adapting ACP communications to meet cultural needs  Complexity of family-based decision-making  Interpreters’ standing in the community | **Knowledge of cultural factors that impact ACP** |
| Physical environment and setting factors   - Physical environment and setting for ACP communication - Power distribution between clinicians and consumes in care delivery setting - Timing for ACP communication | Environmental context and resources | Consultations at home encouraged open communication in ACP  Suboptimal interprofessional collaboration during remote ACP | **The care setting and the physical environment for ACP** |
| Resource related factors   - Availability of ACP resources in non-English languages - Resources for healthcare staff about ACP | Environmental context and resources | Lack of culturally sensitive ACP resources in non-English languages | **Availability of resources to support and conduct ACP** |
